# Reversed Elephant Trunk Technique for the Repair of Type B Aortic Dissection: A Case Report 

**Published:** 2019-04

**Authors:** Anjith Prakash Rajakumar, Mithun Sundararaaja Ravikumar, Karthik Raman, Arun Singh, Ejaz Ahmed Sheriff, Rajan Sethuratnam

**Affiliations:** *Department of Cardiac Surgery, Institute of Cardiovascular Diseases, The Madras Medical Mission, Tamil Nadu, India.*

**Keywords:** *Dissection*, *Aortic aneurysm, thoracic*, *Thrombosis*

## Abstract

We report a case of a type B aortic dissection with an aneurysm treated by the replacement of the proximal descending thoracic aorta via the reversed elephant trunk technique. A 48-year-old asymptomatic man was diagnosed with a type B aortic dissection, moderate aortic regurgitation, and a good biventricular function in March 2012. Four years later (April 2016), a contrast-enhanced computed tomography examination revealed an aneurysmal dilatation in the patient’s descending thoracic aorta with a thrombosis in the proximal part of the false lumen, which warranted surgical repair. He underwent type B aortic dissection repair through the left posterolateral thoracotomy. Three months after the surgery, the patient developed a type A aortic dissection with severe aortic regurgitation, which was successfully managed via a Bentall procedure with arch replacement facilitated by the reversed elephant trunk technique performed during the initial surgery through thoracotomy. At 2 years follow-up, the patient was doing well with a normal left ventricular function.

## Introduction

The reversed elephant trunk technique during the replacement of descending thoracic aortic aneurysms with the arch involvement has previously been described to facilitate a second staged total arch replacement for complex aortic aneurysms involving the entire arch and the descending thoracic aorta.^1^ We describe the reversed elephant technique for the primary treatment of descending thoracic aortic dissections and aneurysms to facilitate a staged total arch replacement if the need arises.

## Case Report

**Figure 1:**
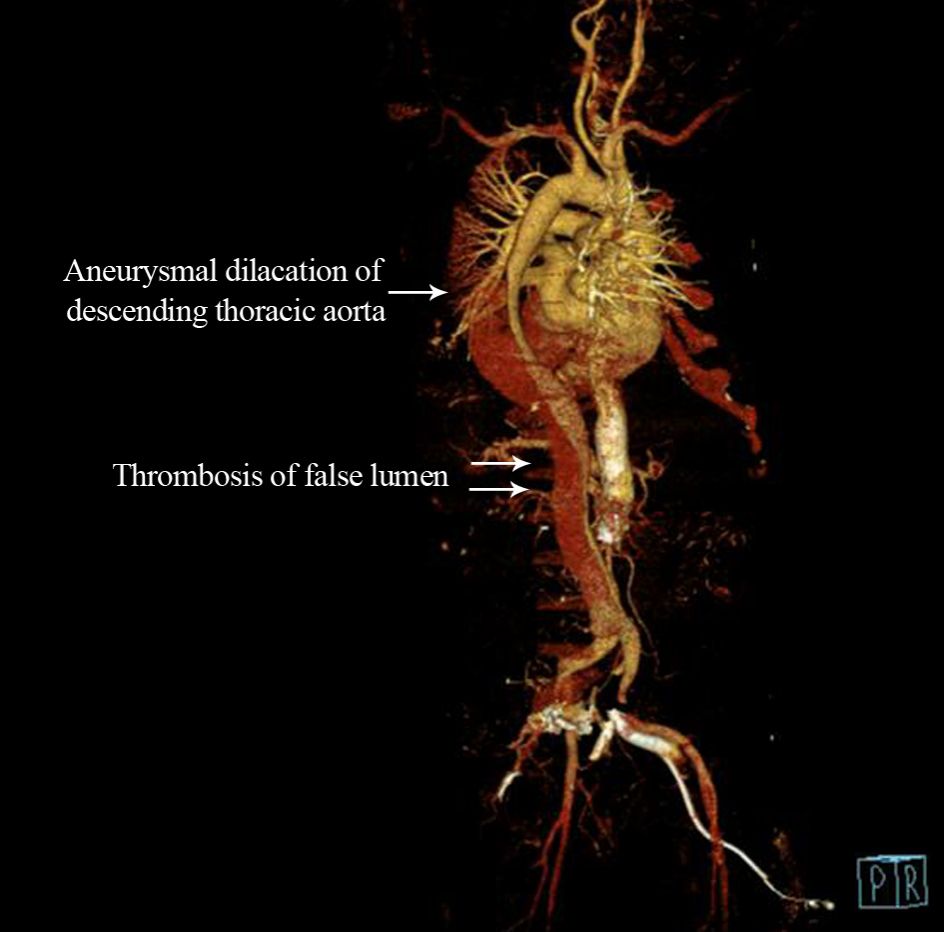
Reconstructed computerised tomography image showing the aneurysmal dilatation of descending thoracic aortic dissection with thrombosis of the proximal part of the false lumen.

A 48-year-old asymptomatic man was diagnosed with a type B aortic dissection, moderate aortic regurgitation, and a good biventricular function in March 2012. A contrast-enhanced computed tomography examination confirmed that the dissection originated distally to the left subclavian artery and traversed into the right common iliac artery. The left kidney was supplied by 2 renal arteries, one of which originated from the false lumen. The patient was managed conservatively with yearly serial echocardiography and computed tomography scan. In April 2016, another contrast-enhanced computed tomography examination revealed an aneurysmal dilatation in the descending thoracic aorta with a thrombosis in the proximal part of the false lumen, warranting surgical repair (Figure 1).

**Figure 2:**
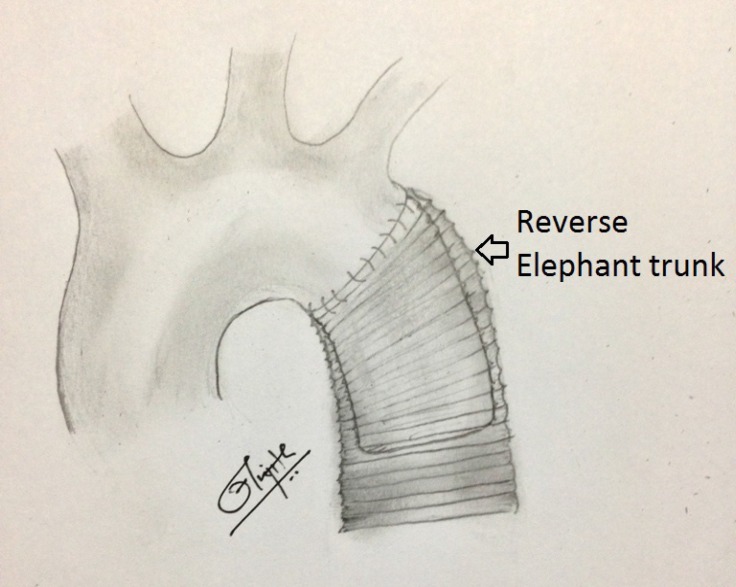
Thoracic aortic aneurysm was replaced with 30 mm interposition Dacron inlay graft using a proximal first technique with a “reversed elephant trunk” left within.

Through a posterolateral thoracotomy in the left fourth intercostal space, cardiopulmonary bypass was instituted following the cannulation of the ascending aorta and the pulmonary artery. A left ventricular apical vent was placed, and the patient was core cooled to 18 ^°^C. The ascending aorta was cross-clamped, and the aortic root cardioplegia was administered. The transverse aortic arch was clamped between the left common carotid artery (LCCA) and the left subclavian artery. The lower thoracic aorta was clamped distally to the aneurysm, and the lower body perfusion was begun with an additional left femoral artery cannulation. The aneurysm sac was opened. The clots and thrombus were evacuated from the false lumen. The true lumen was entered, and the intercostal arteries above the level of T6 were suture closed. The dissection flap was excised, and the aneurysm was replaced with a 30-mm interposition Dacron inlay graft (gelatin-sealed Uni-Graft^®^ graft [B. Braun, Melsungen, Germany]) using a proximal first technique with a “reversed elephant trunk” left within (Figure 2).

Distal anastomosis was performed after fenestrating the septum widely, with it including the intercostals below T6 level. The patient’s postoperative period was uneventful, and transthoracic echocardiography at the time of discharge was satisfactory. 

**Figure 3:**
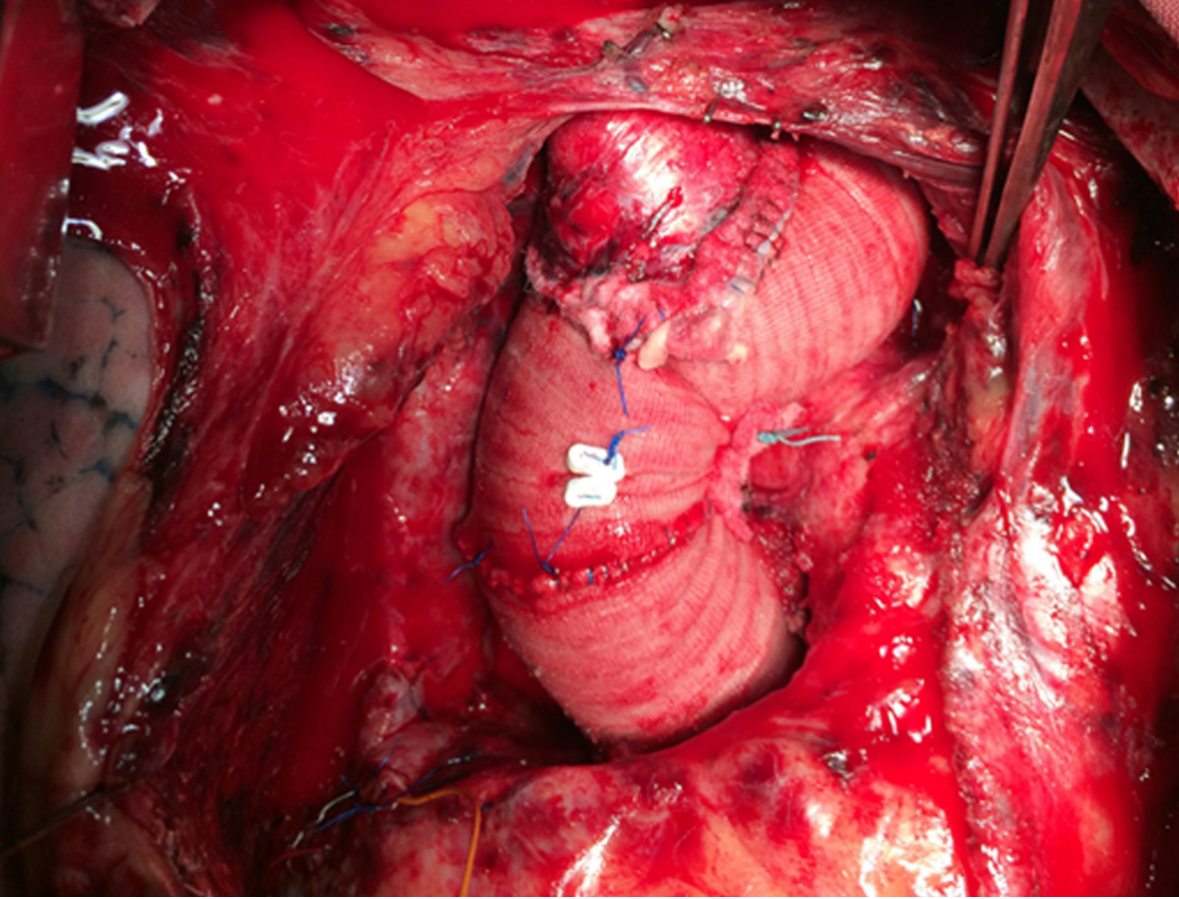
Intraoperative view after Bentall procedure and total arch replacement with Dacron graft with reimplantation of the innominate and the left carotid artery.

**Figure 4:**
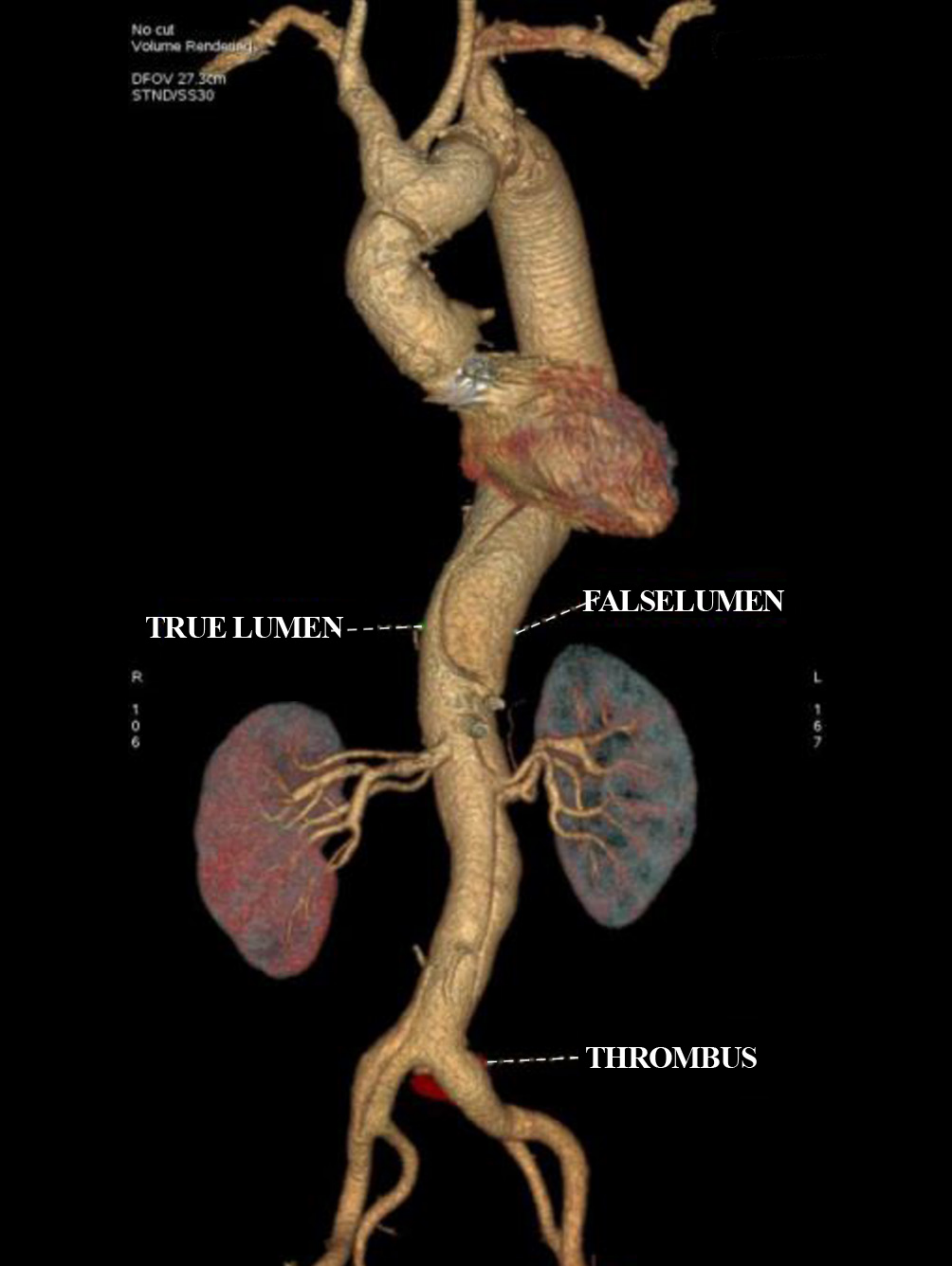
Postoperative CT scan showing a complete replacement of ascending, arch and proximal descending thoracic aorta.

Three months later, the patient presented with acute onset breathlessness of 2 weeks’ duration. Transthoracic echocardiography revealed an acute Stanford type A aortic dissection (TADA) with severe aortic regurgitation and left ventricular dysfunction. He underwent a Bentall procedure with arch replacement using a 28-mm Dacron conduit incorporated with a 25-mm St. Jude aortic bileaflet mechanical prosthesis. Through a median sternotomy, cardiopulmonary bypass was instituted using the right axillary artery and the right atrial cannulation. The arch of the aorta, the innominate artery, and the LCCA were looped. The ascending aorta was cross-clamped, and the left ventricle was vented through the right superior pulmonary vein. The patient was core cooled to 18 ^°^C. An aortotomy was done, and Custodiol^® ^HTK cardioplegia (Sandor, India) was given through the coronary ostia intermittently. The innominate artery and the LCCA were snugged, and selective unilateral antegrade cerebral perfusion was initiated. Bilateral cerebral perfusion was initiated by inserting a 16-F cannula into the LCCA. The intraoperative findings included a TADA involving the right coronary artery and extending into the undersurface of the arch with a severely non-coapting trileaflet aortic valve. The patient underwent a Bentall procedure with arch replacement using the composite conduit. The innominate artery and the LCCA were harvested as an island, and the arch was replaced using a 28-mm Dacron conduit. Distal anastomosis was done onto the previous graft in the descending thoracic aorta after the folded reversed elephant trunk was pulled out, incorporating the left subclavian artery onto the anastomosis. The innominate artery and the LCCA were reimplanted. Next deairing was done, the graft was clamped proximally, and the whole body circulation was re-established. The aortic root was replaced as in the standard Bentall procedure during rewarming. The root/arch replacement was completed by anastomosing the proximal and distal Dacron conduits (Figure 3). The patient was weaned off bypass uneventfully. A computed tomography scan at the time of discharge was satisfactory (Figure 4).

At 2 years’ follow-up, the patient was asymptomatic with a normal left ventricular function.

## Discussion

Patients with a chronic type B aortic dissection (CBAD) have a 5-year survival of 60% to 80% with medical therapy.^2^ An aortic diameter greater than 55 to 60 mm in a CBAD has an estimated 30% risk of rupture per annum. The early mortality rate with thoracic endovascular aortic repair is 6.6% and 8% with open surgical repair (OSR).^2^ OSR is our preferred approach for complicated CBADs. A systematic review by Tian et al.^3^ reported a pooled short-term mortality of 7.5% for OSR in the current era. The authors also reported that the rates of stroke, spinal cord ischemia, renal dysfunction, and reoperation for bleeding were 5.9%, 4.9%, 8.1%, and 8.1%, respectively. In addition, they identified absolute late re-intervention in 11.3% of their patients. According to another investigation, the rate of freedom from re-intervention was 99% in the first year with OSR by comparison with 83% in thoracic endovascular aortic repair.^4^

TADAs following OSR for CBADs are rare. They could be secondary to iatrogenic causes originating at previous surgical sites (aortic cross-clamp, cannulation, or root cardioplegia) or they could arise *de novo*. However, a late manifestation of such an entity is uncommon, as was seen in our patient. The Society of Thoracic Surgeon’s database reports an incidence of 0.06% for aortic dissection as a complication of cardiac surgery.^5^ The management of a TADA with severe aortic regurgitation and the arch involvement in our patient with a Bentall procedure and aortic arch replacement was facilitated by a “reversed elephant trunk” graft left behind in the descending thoracic aorta during a previous surgery for a CBAD (Figure 2). Complicated CBADs after OSR require follow-up for the possibility of TADAs. Leaving a reversed elephant trunk at the proximal anastomosis during OSR for a CBAD is of benefit for the total arch replacement in case a TADA occurs. The reversed elephant trunk technique for the treatment of a mega aorta involving the arch has already been described in the literature.^1^ We propose that this technique be used during the replacement of the descending thoracic aorta itself to facilitate a total arch replacement via median sternotomy at a later stage if the need arises.

## Conclusion

Aortic dissections, especially one involving the whole of the aorta, are always a tricky problem to solve. A multi-staged approach is needed in these types of aortic dissections wherein leaving a reversed elephant trunk proves really beneficial in case the dissection propagates retrogradely and involves the ascending aorta. Type A dissections should be treated surgically on an emergency basis since the waiting period increases the mortality. The reversed elephant trunk forms a good nidus for suturing the distal anastomosis in order to achieve a good hemostatic suture line distally. We recommend this strategy for such types of aortic dissections. 

## Notes:

***This paper should be cited as: ***Rajakumar AP, Ravikumar MS, Raman K, Singh A, Sheriff EA, Sethuratnam R. Reversed Elephant Trunk Technique for the Repair of Type B Aortic Dissection: A Case Report. J Teh Univ Heart Ctr 2019;14(2):81-84.

## References

[1] Fujikawa T, Yamamoto S, Sekine Y, Oshima S, Kasai R, Sasaguri S (2016). Beveled reversed elephant trunk procedure for complex aortic aneurysm. Asian Cardiovasc Thorac Ann.

[2] Hiratzka LF, Bakris GL, Beckman JA, Bersin RM, Carr VF, Casey DE Jr, Eagle KA, Hermann LK, Isselbacher EM, Kazerooni EA, Kouchoukos NT, Lytle BW, Milewicz DM, Reich DL, Sen S, Shinn JA, Svensson LG, Williams DM, American College of Cardiology Foundation/American Heart Association Task Force on Practice Guidelines, American Association for Thoracic Surgery, American College of Radiology, American Stroke Association, Society of Cardiovascular Anesthesiologists, Society for Cardiovascular Angiography and Interventions, Society of Interventional Radiology, Society of Thoracic Surgeons, Society for Vascular Medicine (2010). 2010 ACCF/AHA/AATS/ACR/ASA/SCA/SCAI/SIR/STS/SVM guidelines for the diagnosis and management of patients with thoracic aortic disease. A report of the American College of Cardiology Foundation/American Heart Association Task Force on Practice Guidelines, American Association for Thoracic Surgery, American College of Radiology, American Stroke Association, Society of Cardiovascular Anesthesiologists, Society for Cardiovascular Angiography and Interventions, Society of Interventional Radiology, Society of Thoracic Surgeons, and Society for Vascular Medicine. J Am Coll Cardiol.

[3] Tian DH, De Silva RP, Wang T, Yan TD (2014). Open surgical repair for chronic type B aortic dissection: a systematic review. Ann Cardiothorac Surg.

[4] Fattori R, Cao P, De Rango P, Czerny M, Evangelista A, Nienaber C, Rousseau H, Schepens M (2013). Interdisciplinary expert consensus document on management of type B aortic dissection. J Am Coll Cardiol.

[5] Williams ML, Sheng S, Gammie JS, Rankin JS, Smith PK, Hughes GC (2010). Richard E. Clark Award. Aortic dissection as a complication of cardiac surgery: report from the Society of Thoracic Surgeons database. Ann Thorac Surg.

